# Adaptation towards scale-free dynamics improves cortical stimulus discrimination at the cost of reduced detection

**DOI:** 10.1371/journal.pcbi.1005574

**Published:** 2017-05-30

**Authors:** Wesley P. Clawson, Nathaniel C. Wright, Ralf Wessel, Woodrow L. Shew

**Affiliations:** 1 Department of Physics, University of Arkansas, Fayetteville, Arkansas, United States of America; 2 Department of Physics, Washington University, Saint Louis, Missouri, United States of America; Hamburg University, GERMANY

## Abstract

Fundamental to the function of nervous systems is the ability to reorganize to cope with changing sensory input. Although well-studied in single neurons, how such adaptive versatility manifests in the collective population dynamics and function of cerebral cortex remains unknown. Here we measured population neural activity with microelectrode arrays in turtle visual cortex while visually stimulating the retina. First, we found that, following the onset of stimulation, adaptation tunes the collective population dynamics towards a special regime with scale-free spatiotemporal activity, after an initial large-scale transient response. Concurrently, we observed an adaptive tradeoff between two important aspects of population coding–sensory detection and discrimination. As adaptation tuned the cortex toward scale-free dynamics, stimulus discrimination was enhanced, while stimulus detection was reduced. Finally, we used a network-level computational model to show that short-term synaptic depression was sufficient to mechanistically explain our experimental results. In the model, scale-free dynamics emerge only when the model operates near a special regime called criticality. Together our model and experimental results suggest unanticipated functional benefits and costs of adaptation near criticality in visual cortex.

## Author summary

The cerebral cortex is versatile; depending on changes in behavioral context, the same neural circuit can exhibit dramatically different neural activity and perform different functions. A long-standing hypothesis at the interface of physics and neuroscience posits that such shifts in cortical operation are governed by the same basic principles as those governing phase transitions in certain physical systems. Importantly, this theory predicts changes in information processing as the system changes phases. Here we present experiments on the visual system of turtles, which are consistent with these theoretical predictions. As the system adapts to changes in visual input, we found that cortical dynamics shift towards a scale-free regime, as predicted at the critical point of a phase transition. At the same time, this shift in dynamical regime incurs a tradeoff between sensory detection and discrimination.

## Introduction

Depending on behavioral and environmental context, the same neural circuits can perform different functions. Two essential functions performed by sensory cortices are stimulus detection and discrimination [1]. The ability to *detect* the presence or absence of certain stimuli is crucial when seeking or avoiding aspects of our environment (e.g. food, mates, predators), whereas the ability to *discriminate* among the finer details of sensory input is necessary in many other contexts. Can detection and discrimination be performed simultaneously by the same cortical network or do the two functions require different properties from the underlying circuit?

**Fig 1:**
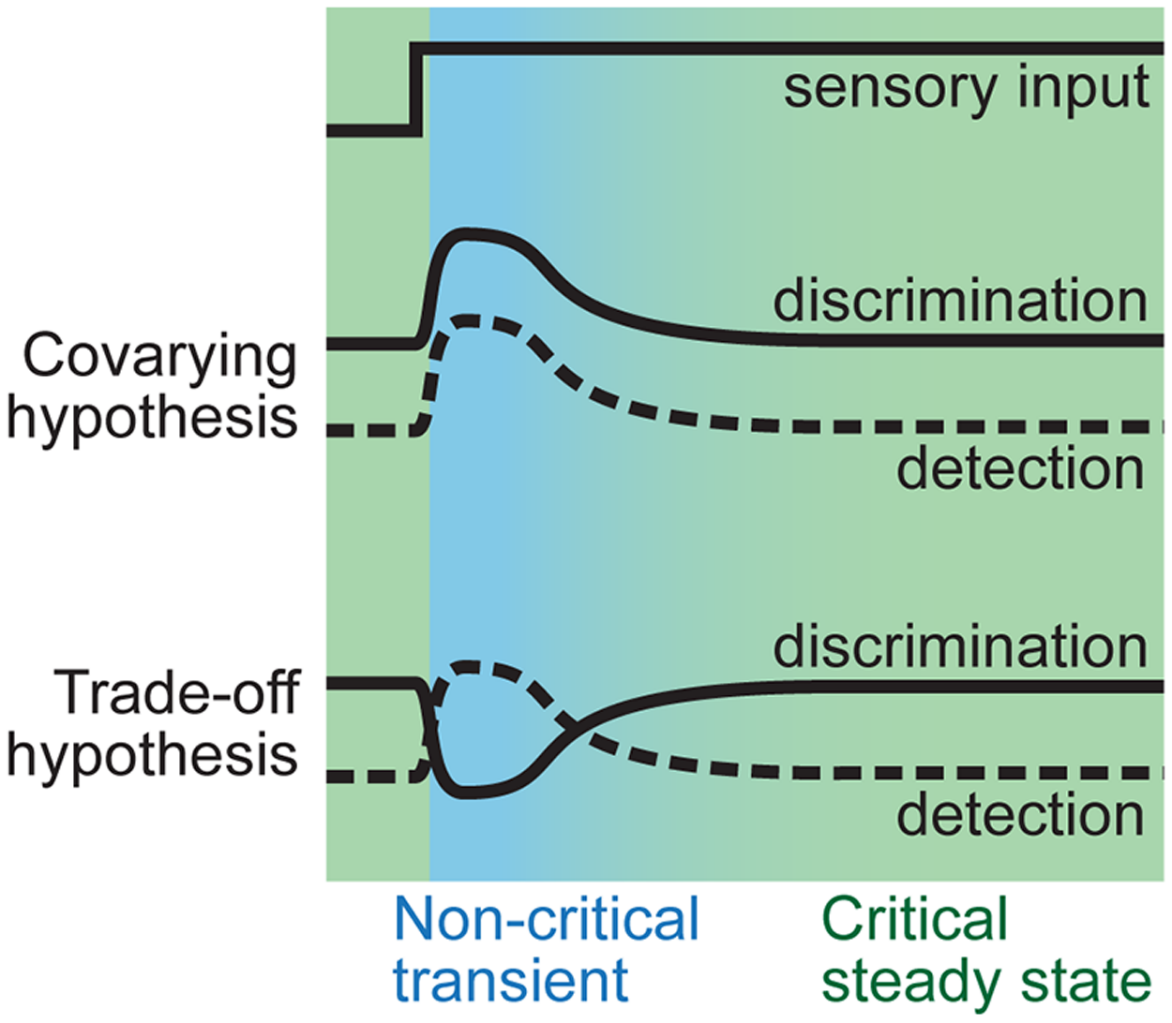
Hypothesized relationships between stimulus discrimination and detection during adaptation. Cartoon illustration of how the gross detection of input may differ from the ability to discriminate fine input differences during adaptation following stimulus onset. One possibility–the covarying hypothesis–is that the highly active transient response carries the most information about the stimulus regardless of whether we consider discrimination or detection. In this view, adaptive depression of synapses reduces information transmission and the critical dynamics of the steady state are too noisy for effective discrimination. Alternatively, the trade-off hypothesis is that the strong onset response is good for detection, but lacks the selectivity needed for good discrimination. This view is in line with the prediction that the critical dynamics of the steady state optimize information transmission.

An important scenario in which this question arises is in visual cortex during adaptation to the onset of a strong visual stimulus [1–3]. In this context, we reframe our question (Fig 1); how do adaptive changes in the cortical network alter the ability of these circuits to detect and discriminate stimuli? Are detection and discrimination better during the transient response or after adaptation has reached a steady-state? Most traditional coding studies do not answer these questions because they have been based on brief (non-adapted) stimuli or, if they considered sustained stimuli, they focused only the steady-state, avoiding the transient response. Studies of the rat somatosensory system suggest that there is a tradeoff [4–7]; as discrimination improves during adaptation, detection worsens, but this remains debated in visual cortex [1,8,9]. In computational models, discrimination can improve or worsen depending on the details of the adaptation mechanisms [10]. Here we test the tradeoff hypothesis in visual cortex of turtles.

To further introduce the tradeoff hypothesis, we compare to some alternative scenarios. One possibility is that stimulus detection and discrimination could vary jointly, both getting worse as adaptation progresses (covarying scenario). In contrast, the tradeoff scenario posits that good discrimination comes at the cost of degraded detection; the two properties change oppositely during adaptation. In both scenarios, detection is most effective during the intense transient response at stimulus onset and decreases as adaptation progresses and response attenuates. How discrimination changes during adaptation is less obvious. In the covarying scenario, the onset response is most informative (for example [9,11,12])–best for discrimination–perhaps because synaptic depression during adaptation lowers spike rates and lowers response information capacity, thereby degrading discrimination. In contrast, the tradeoff scenario supposes that a sufficiently intense onset response can limit information capacity due to saturation and excessive correlations, while adaptation tunes the network into a regime better suited to discrimination [13–15]. Alternative scenarios, intermediate between the two extremes described here, could also exist; for example, discrimination might remain unchanged throughout adaptation (for example [12]).

A separate line of research also predicts the tradeoff hypothesis. Recent experiments [16] and theory [17] suggest that adaptation can tune cortex to a special regime of network dynamics called ‘criticality’. At criticality, network dynamics manifest as diverse spatiotemporal patterns of population activity, governed by specific statistical scaling laws [16,18,19]. For example, the sizes of population activation events are distributed according to a power-law, without a dominant spatiotemporal scale. Throughout this manuscript, we will refer to such power-law distributed population activity as ‘scale-free’. Scale-free dynamics and other features predicted at criticality were previously shown to emerge during adaptation, but were not present during the intense transient response following stimulus onset [16]. Importantly, cortical slice experiments [20] and theory [21–23] suggest that when a network operates near criticality, it is optimal for stimulus discrimination, although these studies did not address adaptation. Similarly, Fisher information is predicted to peak at criticality [24]. Thus, consistent with the tradeoff hypothesis, these studies predict the emergence of criticality and enhancement of discrimination during adaptation, but this prediction has yet to be tested.

Here our study was designed with two goals (Fig 1). First, we aimed to determine whether there is an adaptive trade-off between detection and discrimination or whether the two functions co-vary. Second, we sought to link adaptive changes in these two functions to changes in cortical network dynamics. We found that changes in network dynamics were consistent with adaptation tuning the cortex to a steady state near criticality after a transient response that was far from criticality. Discrimination was higher when the network was near criticality, while detection was higher during the non-critical transient. Thus, our results confirm the tradeoff hypothesis in visual cortex and associate a functional tradeoff with changes in cortical state near criticality.

## Results

**Fig 2:**
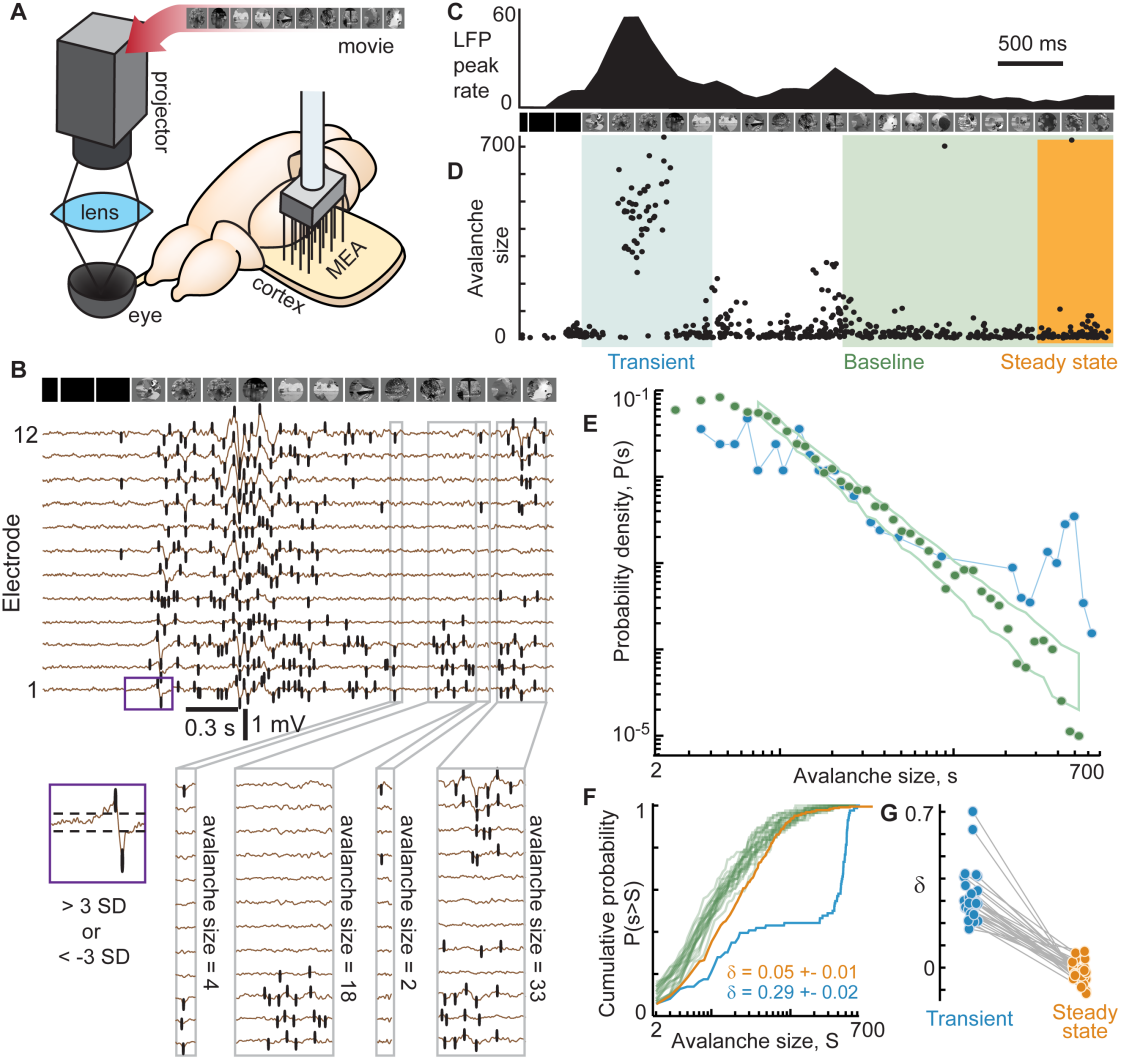
Adaptation tunes cortical dynamics from large-scale transient response to scale-free steady-state. (**A**) Motion-enhanced movies were projected onto the retina while recording local field potential (LFP) with a microelectrode array in visual cortex of the turtle *ex vivo* eye-attached whole-brain preparation. (**B**) Shown are LFP traces from a subset of 12 electrodes. Intense population activity occurs at the onset of the movie; adaptation leads to more moderate steady-state activity. To characterize these changes in population activity, we analyze ‘avalanches’ which are defined as spatiotemporal clusters of LFP peaks beyond ±3 SD (black ticks). Avalanche size was defined as the number of LFP peaks comprising the cluster. (**C**) LFP peak rate time series, averaged over 80 movie repetitions. (**D**) Each point represents the size and time (middle of duration) of one avalanche. Avalanches from 80 trials are overlaid. Avalanches were typically very large during the transient response, but adaptation resulted in smaller and more diverse sizes. (**E**) Typical distributions of avalanche sizes during the transient (blue, 0–1 s after movie onset) and the baseline period (green, 2–5 s after movie onset). During the transient, the distribution exhibited a ‘bump’ at large size indicating a high likelihood of very large avalanches. During baseline, avalanche size distributions were well-described by a power-law function, in line with recent findings that adaptation tunes cortical network dynamics to criticality. Green box delineates the expected range (5–95 percentile) of probabilities for a finite sample drawn from a perfect power law. (**F**) To quantitatively characterize population dynamics during different time periods we computed δ, which measures deviation from the baseline distribution. Calculation of δ is based on differences between cumulative distributions like the examples shown. (**G**) Shown is a summary of 14 experiments. Without exception, the transient exhibited many more large avalanches than the baseline (δ>0) while the steady state period (4–5 s after movie onset) exhibited small deviations, both positive and negative, from baseline.

To investigate adaptive changes in dynamics and function of cortical networks, we employed a visual stimulation paradigm that entails strong adaptation in the visual system. We suddenly switched from no visual input (darkness) to a movie projected onto the retina of the ex vivo turtle eye-attached whole-brain preparation [16,25] (Fig 2A). Using a microelectrode array we recorded local field potential (LFP) and multi-unit activity (MUA) from visual cortex (Fig 2B). We observed intense neural response during a brief transient following movie onset and attenuated response as the system adapted towards a steady-state during continued movie stimulation (Fig 2C). In the following sections, we first show how collective population dynamics change during adaptation, based on analysis of LFP. Then, we delineate adaptive changes in visual detection and discrimination, based on both LFP and MUA. Next, we show that these adaptive changes in function are not the same in all animals and that variability in population dynamics is correlated with variability in function. Finally, using a network-level computational model, we demonstrate that short-term synaptic depression provides a simple explanation of our experimental findings.

### Adaptation results in scale-free cortical network dynamics

To characterize adaptive changes in cortical network dynamics, we examined spatiotemporal bouts of large amplitude, correlated LFP fluctuations, called neuronal avalanches [16,20,21,26,27]. Briefly, a neuronal avalanche was defined as a temporally correlated cluster of LFP peaks, often occurring on many electrodes (Fig 2B, Methods). Avalanche size was defined as the number of constituent LFP peaks (Fig 2B). After repeating the visual stimulation paradigm many times (n = 80), we combined all avalanches that occurred during transient periods (0–1 s after stimulus onset) into one group and, in a separate group, we combined all avalanches that occurred in a later period (2–5 s after stimulus onset), labeled the ‘baseline’ period in Fig 2D. Comparing probability distributions of avalanche sizes from the transient versus the baseline provided concise and quantitative characterizations of how cortical network dynamics change during adaptation.

During the transient period, we found that avalanche size distributions were bimodal in shape, indicating the tendency for large avalanches often spanning the entire recording area (Fig 2E). In contrast, as the visual cortex adapted towards a steady-state regime, avalanche sizes became more diverse with a scale-free size distribution, i.e. the size distribution became power-law in shape (Fig 2E). Using statistically rigorous methods [16], we found that avalanche sizes during the baseline period were power-law distributed (significance parameter q = 0.2 ± 0.26, see Methods), with exponents -1.8 ± 0.3, consistent with previous findings [16]. Importantly, such scale-free avalanche distributions are expected when a network operates at criticality, while bimodal distributions are inconsistent with criticality. Thus, in line with previous work [16], our results are consistent with the conclusion that the onset of stimulation drives the visual cortex into a state far from criticality and adaptation tunes the system towards criticality.

One interesting implication of this finding is that the spatiotemporal changes in visual input during the movie could also cause moment-to-moment deviations from scale-free dynamics. Indeed, the onset of the movie is simply a particularly intense change in visual input. Deviations from scale-free dynamics during the movie could be ‘averaged out’ when considering the entire baseline period (2–5 s after stimulus onset). Therefore, to test this possibility, we examined avalanche size distributions during a shorter time period well after the transient (4–5 s after stimulus onset), labeled ‘steady-state’ in Fig 2D. For both the steady-state periods and the transient periods, we computed the deviation δ from the baseline avalanche size distribution, based on summed differences between cumulative distributions, similar to a Kolmogorov-Smirnov statistic (Fig 2F; Methods). As expected, the deviation δ was large during the transient (0.32 ± 0.12, mean ± SD) and near zero in the steady-state (-0.02 ± 0.04; Fig 2F). However, there was substantial brain-to-brain variability in δ for the steady-state period.

To interpret the meaning of this variability in δ for the steady-state period, we note that the steady-state time period was deliberately chosen to overlap with the baseline time period. Thus, the steady-state distribution is based on a subset of all the avalanches that occur during the full baseline period. A non-zero deviation δ in the steady-state indicates a brief excursion from the time-averaged statistics of the baseline period. Experiments with positive values of steady-state δ suggest a brief excursion towards large-scale dynamics like those observed during the transient. Experiments with negative values of δ may indicate an excursion towards small-scale dynamics, consistent with a somewhat subcritical regime, in which large avalanches are relatively rare (further discussion of this possibility is in the model results below). In the following section, we will show that these variations in network dynamics revealed by δ are correlated with variations in how visual cortex processes input.

### Improved stimulus discrimination at the cost of reduced detection

Next we sought to relate the adaptive changes in network dynamics described above to changes in how the cortex encodes the sensory input. We focused on two important aspects of cortical coding, stimulus detection and stimulus discrimination (Fig 1).

**Fig 3:**
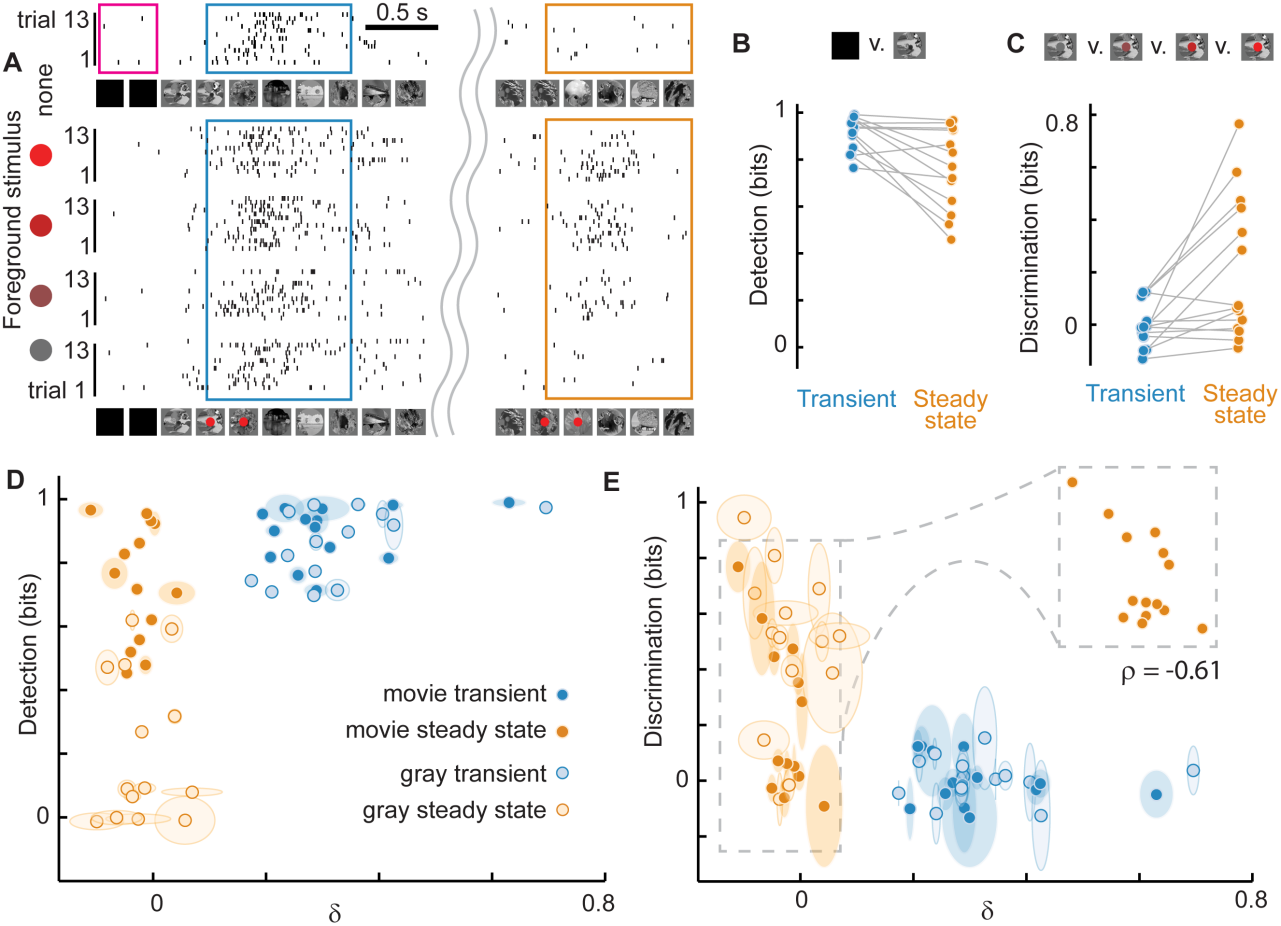
Adaptation enhances discrimination at the cost of reduced detection: LFP rate coding. (**A**) TOP: Typical rasters of LFP peaks (taken from all electrodes, randomly subsampled to 10%) showing response to 13 trials with the same background movie visual stimulus (no foreground). Detection was quantified based on the LFP peak count when the movie was off (pink box) compared to when the movie was on (blue or orange box). BOTTOM: For discrimination a foreground stimulus (4 different red dots) was presented during each of four blocks with 13 trials each, all with the same background movie stimulus. The red dot was presented either during the transient period just after background stimulus onset (blue box) or later during the steady-state (orange box). Note that the different foreground stimuli were more easily distinguished by the LFP peaks in the steady-state compared with the transient, but the presence of the background stimulus is more easily detected based on strong transient response. (**B**) Summary (n = 14 turtles) of how well the LFP peak count can detect the presence of the background stimulus. All turtles show a decrease in detection from transient to steady-state. (**C**) Summary of how well the LFP peak count can discriminate the four different foreground stimuli. Most turtles showed an increase in discrimination from transient to steady-state. (**D, E**) A more refined explanation of detection and discrimination is obtained by comparing to δ. Generally, lower δ resulted in enhanced discrimination and poorer detection, while higher δ exhibited the opposite trend. Thus, power-law distributed (low δ) population dynamics are associated with a functional trade-off, gaining discrimination at the cost of decreased detection. The inset is an expanded view to better show the correlation between discrimination and δ during the steady-state.

First, we measured adaptive changes in detection. We took the perspective of an ideal observer and asked to what extent the presence of the movie was detected based on the LFP cortical population response. We answered this question first based on activity recorded during the transient and then based on activity during the steady state (Fig 3A). To quantify stimulus detection, we calculated the mutual information I(R;S) of visual stimulus and neural response, adjusted for low sample count bias (see Methods). The stimulus set S was binary, consisting of many repetitions (n = 80) of two possible stimuli–movie off or movie on. The response set R was defined as the LFP peak count during a 1 s period while the movie was off or on. The ‘movie on’ response was taken from either the transient period or the steady state period, thus allowing us to compare the efficacy of detection during these two different stages of adaptation. We found that stimulus detection was high during the transient period (0.9 ± 0.1 bits) and typically reduced during the adapted and critical steady state (0.7 ± 0.2 bits; Fig 3B).

Next, we measured adaptive changes in discrimination. Here, our goal was to go beyond the rather coarse and simple detection considered above and assess how more detailed information about the visual input is represented in the cortex. To meet this goal, we modified the visual stimulation paradigm described above. We presented the same movie stimulus many times (n = 80), but now with four different ‘foreground’ stimuli, each presented 20 times in pseudorandom order, superimposed on the ‘background’ movie. The foreground stimulus was a red dot with four different levels of ‘redness’ ranging from gray to bright red (Fig 3A). This foreground red dot was presented either during the transient immediately following movie onset or later during the steady state. As with detection, we quantified discrimination using the mutual information I(R;S) of stimulus and response. However, the stimulus set S was different, now representing the four possible foreground red dot stimuli. Response was defined as the LFP peak count during a 1 s period immediately following the red dot presentation (Fig 3A). Stimulus discrimination was low during the transient period (-0.01 ± 0.1 bits) and higher in the adapted and critical steady state (0.2 ± 0.3 bits; Fig 3C).

**Fig 4:**
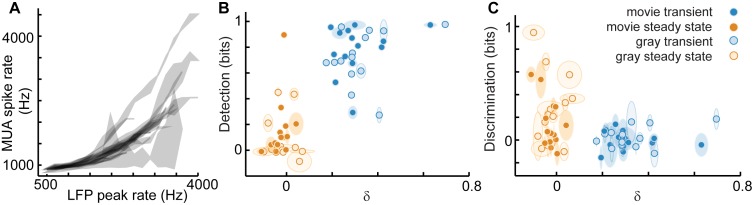
Adaptation enhances discrimination at the cost of reduced detection: MUA rate coding. (**A**) Based on the entire recording for each turtle, the LFP count per 1 second time window is strongly correlated with the MUA spike count per 1 second. MUA was generally lower rate than LFP. Each shaded region corresponds to one experiment. The vertical extents of the shaded regions delineate quartiles. (**B**) Similar to our observations based on LFP peak response (Fig 3), we found that when response was defined based on MUA spike counts, detection is highest during the transient response and decreases as adaptation progresses to a steady state. The decrease in detection during the steady-state compared with LFP-based detection is likely due to the lower activity rate of MUA relative to LFP. (**C**) MUA spike response also exhibited increased discrimination during the steady state compared with the transient.

Next we performed the same detection and discrimination analysis, but using MUA spike counts instead of LFP peak counts to define response. First we note that, in general, MUA spike rate is strongly correlated with LFP peak rate (Pearson’s ρ = 0.8 ± 0.2, p<10^−4^, p value based on permutation distributions, Fig 4A). Next, we computed detection and, like the LFP-based results, we observed that detection is strong in the transient response (0.8 ± 0.2 bits) and relatively weak in the steady state response (0.2 ± 0.2 bits) (Fig 4B). Likewise, discrimination improved from 0.001 ± 0.1 bits during the transient to 0.1 ± 0.2 bits during steady state (Fig 4C).

Thus, we conclude that, in visual cortex, there is an adaptive tradeoff between stimulus detection and discrimination during adaptation; detection drops from an initially high level while discrimination improves. To test whether these findings generalized beyond stimuli with naturalistic spatiotemporal structure, we also considered adaptation following the onset of a static gray screen, instead of a movie. The conclusions were largely the same as for the movie stimuli (Figs 3D, 3E, 4B and 4C).

In the preceding results, we have shown that both network dynamics and function evolve systematically over the time course of adaptation. The correlation between network dynamics and function becomes more apparent when we plot detection versus δ (Fig 3D) and discrimination versus δ (Fig 3E). Discrimination was anticorrelated with δ (for LFP ρ = -0.6, p<10^−5^; for MUA ρ = -0.38, p<0.004) and detection was correlated with δ (for LFP ρ = 0.6, p<10^−5^; for MUA ρ = 0.8, p<10^−13^). More interesting, we found that, even within the steady state period alone, variability in network dynamics, i.e. variability in δ, could explain a significant amount of the variability in discrimination (for LFP ρ = -0.6, p<0.02, Fig 3E; for MUA ρ = -0.56, p<0.03, Fig 4C). Such brain-to-brain variability in discrimination at the same time point following movie onset (4–5 s after movie onset) highlights the fact that the same movie background stimulus caused different neural response in different brains. Thus, presumably, differing degrees of adaptation were present in different brains. Moreover, the fact that stimulus discrimination improved with decreasing δ values, including into the δ < 0 regime, suggests that discrimination of foreground stimuli is optimal when network dynamics deviate from scale-free, towards the small-scale. In the context of the criticality hypothesis, this would indicate that slightly subcritical dynamics are better for discrimination than criticality.

**Fig 5:**
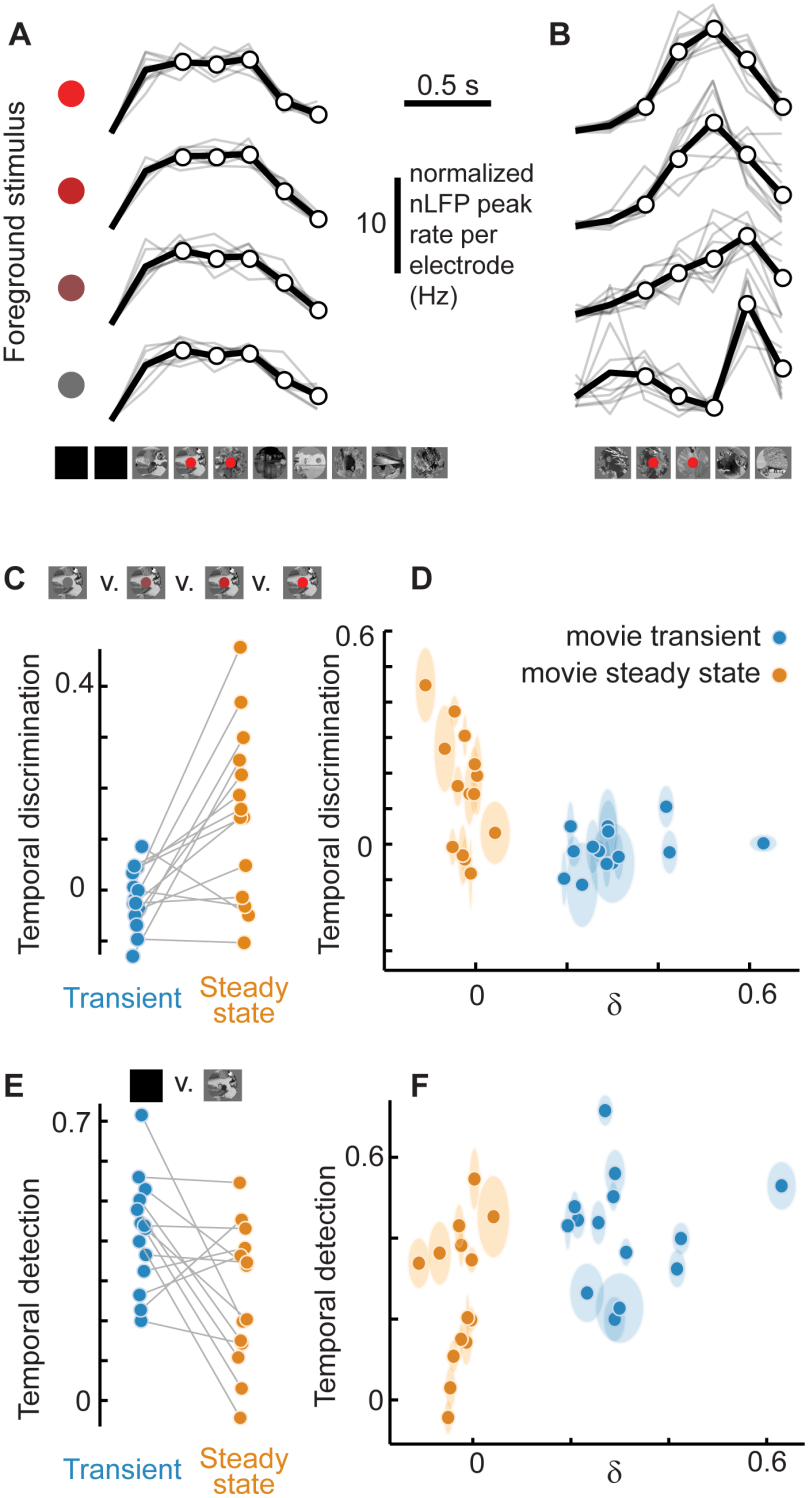
Adaptation enhances discrimination at the cost of reduced detection: Temporal coding. (**A**) The time course of population response during the 1 s (0.2 s resolution) following the four different foreground stimuli (red dots) showed little difference during the transient response. Black lines indicate response averaged over repeated trials. Gray lines indicate individual trials. Each response was subtracted by its time average and normalized by its variances to emphasize effects of rate coding. One example turtle shown. White dots indicate the 5 bins used to compute the 5 bit temporal response. **(B)** During the steady-state, different foreground stimuli evoked differing temporal structure of responses. Thus, temporal structure carries useful information for discrimination. **(C)** Summary of discrimination based on temporal structure for 14 experiments. Most turtles showed an increase in discrimination from transient to steady-state. **(D)** Variability in discrimination is better explained when changes in δ are accounted for. Similar to rate-based discrimination, power-law distributed (low δ) population dynamics are associated with optimal temporal discrimination. **(E)** Summary of detection based on temporal structure for 14 experiments. Detection typically decreased from transient to steady-state. **(F)** Variability in detection is better explained when changes in δ are accounted for. Similar to rate-based detection, power-law distributed (low δ) population dynamics are associated with low detection.

In the above analysis, detection and discrimination were calculated assuming a rate based code (LFP peak rate in a fixed time window). Previous studies highlight the possibility that temporal or spatial patterns can encode information in addition to that encoded in rates [28–31]. Therefore, we next sought to determine if other forms of coding also exhibit an adaptive tradeoff between detection and discrimination. We tested this possibility for a temporal code and a spatial pattern code. First, we computed discrimination based on a 6-bit spatial pattern of response (normalized to remove dependence on activity rate.) Each bit represented a different, spatially contiguous group of electrodes in the matrix array. We found that spatial pattern of response did not carry significant information for discrimination of the foreground red dot stimuli. However, we next tested the temporal pattern of response and found adaptive improvement of discrimination based on such temporal coding. We defined the temporal response to a single stimulus to be a 5-bit binary ‘word’ corresponding to a sequence of 5 consecutive time bins, each 0.2 s in duration, following the onset of the foreground red dot (Fig 5A and 5B). Each bit was set to 1 if its LFP peak count (summed over all electrodes) was higher than the mean across the 5 time bins (counts were also normalized by variance across time). By this definition, the temporal pattern of response is normalized to emphasize temporal aspects of the response rather than the rate coding considered above (but may not be entirely independent of rate as discussed elsewhere [31]). We computed mutual information between this 5-bit temporal response and the stimulus (foreground red dot levels), corrected for low sample bias as done with the rate coding discrimination presented above. We found that the temporal pattern of response often did carry information about the stimulus, but only during the steady-state (0.15±0.17). During the transient response, temporal information was minimal (-0.01±0.06, Fig 5C). Moreover, temporal discrimination was significantly anticorrelated with changes in network state as measured by δ (ρ = -0.45, p<0.005). We note that when unstructured, static background stimulation (gray screen) was used instead of the movie background, adaptive improvement of temporal discrimination was less prominent and only observed in a minority of turtles. Comparing among the 9 turtles with significant discrimination during the steady-state, 6 had higher rate-based discrimination compared with timing-based discrimination. To determine if rate and timing were redundant or carried different information, we next computed discrimination using both rate and timing responses (joint mutual information). We found that this joint-discrimination was close to the individual values of rate-discrimination and timing-discrimination (difference of 0.04 ± 0.14 bits), which suggests that the rate and timing information was rather redundant. In contrast, if the rate and timing information was not redundant, i.e. coded different aspects of the stimulus, we would expect the joint-discrimination to be closer to the sum of the rate-discrimination and timing-discrimination. This difference was 0.36±0.24 bits. In summary, adaptive improvements of discrimination were most obvious when considering a rate code, but generalize beyond a simple rate code and apply to temporal population coding as well.

We also considered whether temporal response pattern is better for detection during the transient response than in the steady-state, as we found for rate-based response. First, compared to rate based detection, we found that detection based on temporal response was poor (but far from zero). This is perhaps not surprising since, during darkness, there is no expectation for temporally structured activity to encode the lack of stimulus. Nonetheless, there was a small, but significant decrease in detection from the transient to the steady state. Detection using temporal response was 0.42 ± 0.14 bits during the transient and 0.26 ± 0.17 bits during the steady state (Fig 5E and 5F).

### Synaptic depression can mediate the computational trade-off

**Fig 6:**
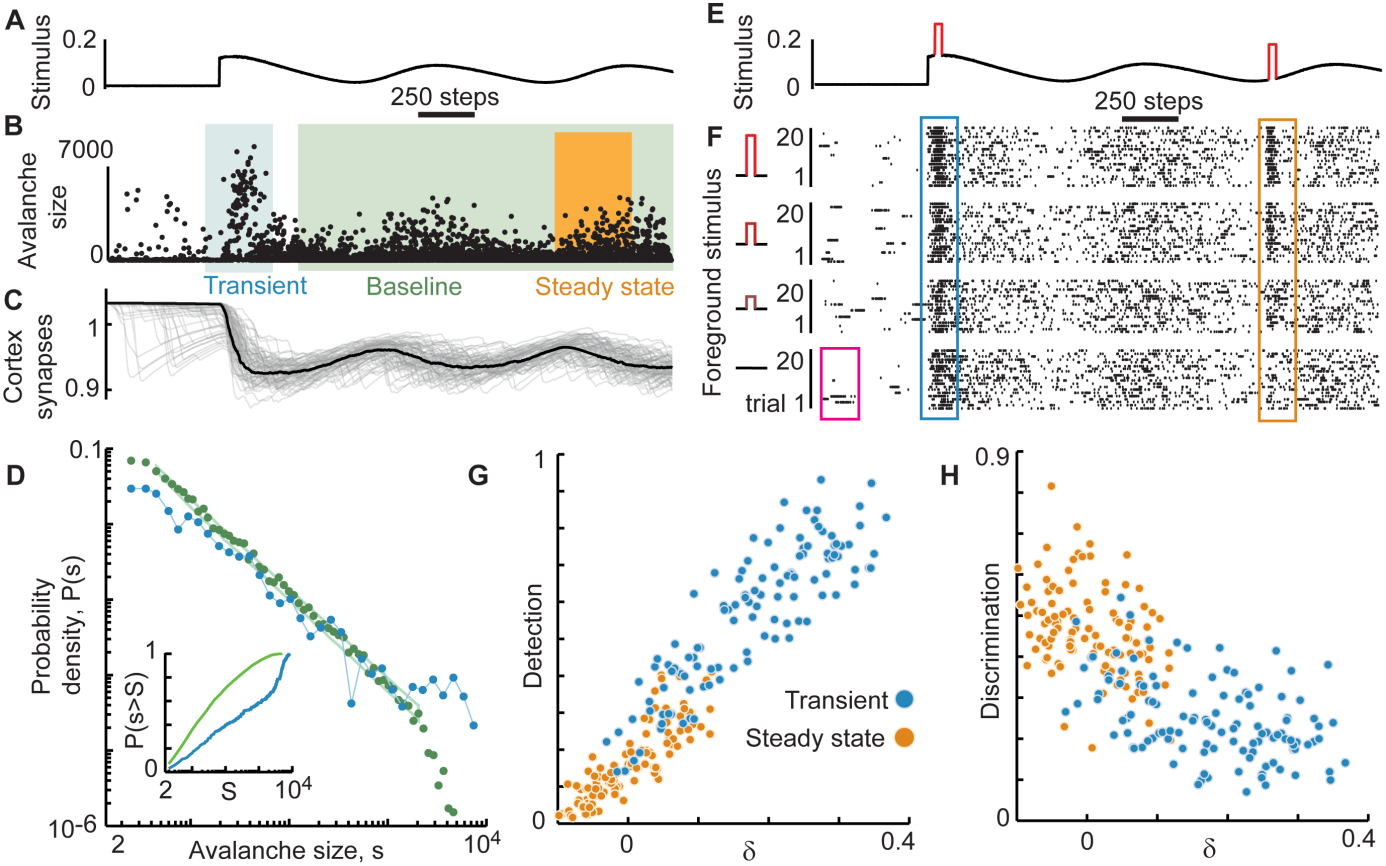
A network model with short-term depression reproduces the discrimination-detection trade-off. (**A**) The model network is driven by a slowly varying ‘background’ stimulus that turns on at t = 500 timesteps. We interpret 1 time step to be approximately 1 ms. (**B**) Avalanches with a tendency for very large sizes occur during the transient following stimulus onset (blue). Smaller avalanches occur during the baseline period (green), which includes the later time period labeled ‘steady-state’ (orange). Avalanches are overlaid from 80 repetitions of the same background stimulus. (**C**) Synapse strength (averaged over all except the input synapses) drops at stimulus onset and fluctuates due to short term depression. Gray lines indicate single trials (n = 80); black line is the cross-trial average. (**D**) Avalanches are distributed according to a power-law during the baseline (green) and have a high likelihood of very large avalanches during the transient (blue). Inset: cumulative distributions of the same data reveal that δ>0 for the transient. (**E**) A ‘foreground’ stimulus (red) is applied at two times: during the transient and later during the steady-state. (**F**) Raster of model spikes (from all neurons, subsampled) including 80 trials, broken into four blocks of 20, each with a different intensity of foreground stimulus. Response was defined as the spike count during the transient (blue) or the steady-state (orange). (**G, H**) Consistent with our experiments, discrimination of foreground stimuli was inversely proportional to δ while detection was proportional to δ.

What biophysical mechanisms could explain our observations of a trade-off between detection and discrimination as a network adapts from large-scale transient response towards scale-free dynamics (Figs 3 and 4)? Previous studies suggest that short-term synaptic depression is sufficient to explain adaptation towards criticality, where scale-free dynamics are expected [16,17]. Can such a simple mechanism also account for the observed trade-off between detection and discrimination? We investigated this possibility in a model network of excitatory and inhibitory probabilistic integrate-and-fire neurons with all-to-all connectivity and short-term synaptic depression [16] (Methods). To mimic the experimental visual stimuli (movie onset), the model was subjected to an abrupt increase in input rate followed by a slowly varying input rate (Fig 6A). This process was repeated 80 times. As in the experiments, we examined three time periods during the time course of adaptation following the onset in background stimulation–the transient, baseline, and steady state (Fig 6B). As found experimentally (Fig 2E), extremely large avalanches were common during the transient period (Fig 6B). Avalanches decreased in size as synapses depressed (Fig 6C). Specifically, the avalanche size distribution during the transient period displayed a distinct bimodal character, while a power-law distribution emerged as adaptation progressed into the baseline period (Fig 6D), consistent with the hypothesis that synaptic depression can tune the cortex towards criticality.

Deviations from the baseline power-law were large during the transient (δ = 0.18 ± 0.1). The steady state test period exhibited much smaller deviations from the baseline power-law (δ = 0.004 ± 0.06). Thus, the effects of synaptic depression on the model network dynamics matched well the adaptive changes in network dynamics we observed in our experiments (Fig 2F). We next investigated the impact of synaptic depression on detection and discrimination in the model. We focused on a rate code, since the majority of discrimination information in the experimental data was carried by a rate code.

Mirroring the experimental approach, we asked to what extent the presence of the elevated external input (Fig 6E) was detectable based on the simulated network spiking (Fig 6F) and how this detection was affected by synaptic depression. Detection was computed the same way as in the experimental data analysis presented in (Figs 3 and 4), with response defined in terms of spike count. For all networks and trials tested, stimulus detection was high (0.57 ± 0.19 bits) during the transient period at stimulus onset and significantly reduced (0.17 ± 0.10 bits) during the adapted and critical steady state. In this respect, the model reproduced the adaptive changes in stimulus detection in turtle visual cortex.

The model also successfully reproduced the dynamics of stimulus discrimination we observed experimentally. We asked to what extent four levels of a brief increase in external input rate (foreground stimulus) on top of the high external input rate (background stimulus) were discriminated based on the simulated network spiking (Fig 6E and 6F). For all networks and trials tested, stimulus discrimination was low (0.25 ± 0.10 bits) during the transient period at stimulus onset and higher (0.46 ± 0.11 bits) during the adapted and critical steady state. Thus, we conclude that short-term synaptic depression is sufficient to reproduce our experimentally observed tradeoff between detection and discrimination.

Our experiments exhibited substantial brain-to-brain variation in discrimination and detection (Figs 3B, 3C, 4B and 4C). This functional variability was partially explained by accounting for variability in the network state (the δ measure) (Figs 3D, 4E, 4B and 4C). As discussed above, one possible explanation of these variations in network state and function is that the same visual stimulus could result in a different time course of population response in different animals, and therefore, a different time course of adaptation. To test this idea in our model, we systematically varied the time course of the slowly varying component of the background stimulus (Fig 6A). We temporally shifted the peaks and valleys of the background stimulus with respect to the foreground stimulation onset and computed δ during the same transient and steady state time periods. This resulted in a range of δ from approximately 0 to 0.4 for the transient period and from approximately -0.1 to +0.1 in the steady state period. As found experimentally, discrimination in the steady state time period was anticorrelated with δ. Discrimination was highest when the system exhibited nearly scale-free dynamics, but slightly shifted towards small-scale (δ < 0) (Fig 6H), consistent with a slightly subcritical state.

**Fig 7:**
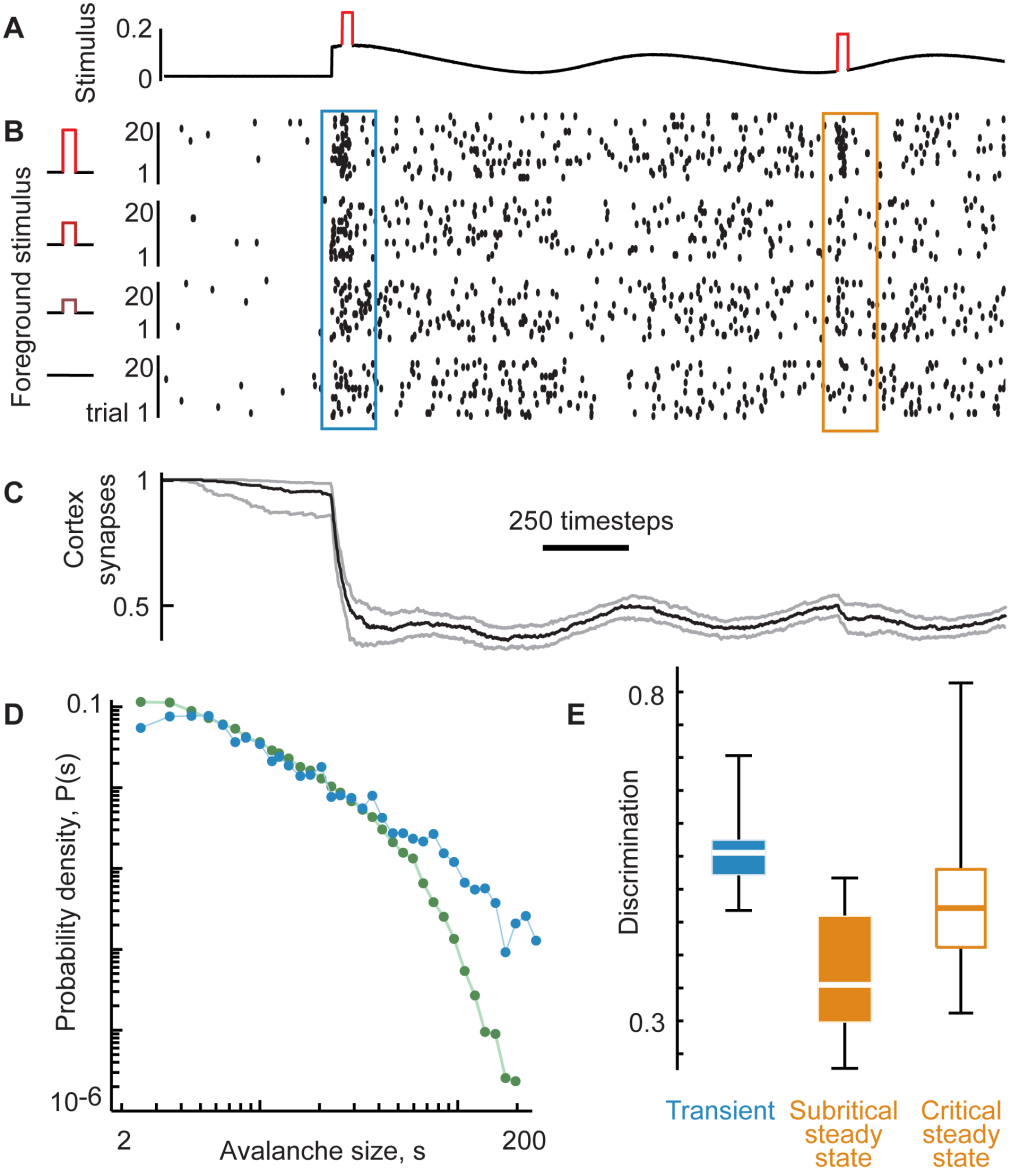
Extreme synaptic depression results in small-scale dynamics and decreased discrimination. (**A**) Shown are results from our model with more extreme depression with *τ*_*d*_ decreased by a factor of 100 compared to the model results in Fig 6. All other model parameters including the stimulus paradigm are unchanged. (**B**) Subsampled spike rasters for 20 trials for each of 4 different foreground stimulus levels. Note that the steady state spike rate is lower than that in the model results of Fig 6. Spike response does not vary strongly with changing foreground during the steady state. (**C**) The extreme depression implemented here results in about 50% reduction in cortex synapse strengths. This is a large reduction compared to the ~10% reduction for the model results in Fig 6. (**D**) During the baseline period (same definition as shown in Fig 6), avalanche sizes are not distributed according to a power-law (green), indicating that the dynamics are not at criticality. Rather, the curvature of the distribution and the lack of large avalanches are consistent with subcritical dynamics. In contrast, the avalanche size distribution during the transient is close to a power-law because the synapses have not yet reached a low enough level to strongly deviate from criticality. (**E**) In contrast with the results in Fig 6, the transient period (blue) exhibits high discrimination as expected for critical dynamics. The subcritical steady state (solid, orange) exhibits decreased discrimination compared with the critical steady state of Fig 6 (open, orange). Box vertically spans the two quartiles around the median (middle line). Whiskers indicate the range of the data.

These observations raise a question: if we push the cortex further towards small-scale dynamics (further subcritical) does discrimination continue to improve, or does discrimination drop in more extreme regimes? We used our model to answer this question (Fig 7). We made depression stronger (100 times stronger than the results in Fig 6) and the model steady state displayed extremely subcritical network dynamics, strongly deviating from scale-free avalanche sizes (Fig 7D). For such extreme synaptic depression, stimulus discrimination declined (Fig 7E). Together with our experiments (Figs 3E and 4C) and other recent model studies [22,32], this is consistent with the hypothesis that there is an optimal network state for stimulus discrimination near criticality, but slightly subcritical.

## Discussion

Cortical neural circuits are computationally versatile, capable of functionally reorganizing themselves to accommodate changes in sensory input. How two specific functions–stimulus detection and discrimination–emerge from the collective dynamics of the same neural circuit has remained an important and unanswered question [4–7]. Here, we found, using experiments and computational modeling, that adaptation to a change in sensory input incurred a shift in cortical dynamics and a trade-off between visual detection and discrimination. Visually-driven cortical dynamics shifted from an initially intense large-scale response to a more moderate, scale-free network dynamics, consistent with recent findings that adaptation tunes visual cortex towards criticality [16]. Concurrently, we found that adaptation towards scale-free dynamics coincided with enhancement of stimulus discrimination at the expense of decreased stimulus detection. Our simulations of a model network demonstrated that short-term synaptic depression can provide a mechanistic explanation for the computational trade-off during network adaptation towards criticality.

Our study offers an answer to an important, but typically overlooked, question. How do adaptive changes at the synaptic or single-neuron level impact network-level dynamics? It is well known that changing single neuron properties [33] or altering synaptic interactions [34] can result in dramatic changes in the collective dynamics of the cortical network. Moreover, many aspects of cortical function are sensitive to such changes in cortical state [33–35]. Given that adaptation can alter single neuron properties and synapses, it is important to determine how adaptation impacts cortical network dynamics. Traditional theoretical studies have focused on how adaptation impacts detection and discrimination at the single neuron, or single synapse level. Some of these studies even predicted a tradeoff between detection and discrimination, e.g. [36]. However, these studies left open the possibility that the observed neuronal or synaptic changes entail changes in large-scale network dynamics which, in turn, feed back and disrupt the conclusions found at the small scales. For example, most theoretical single neuron studies assume a certain level of background noise to account for the network input to the neuron. The assumption that such background noise is independent of single-neuron adaptive processes could lead to erroneous conclusions if adaptation changes the nature of the background network dynamics. Our results suggest that by tuning visual cortex towards a scale-free dynamical regime, adaptation facilitates improvements in discrimination.

Previous experiments [4–7] on mammalian somatosensory cortex observed a trade-off between detection and discrimination, similar to that we found in turtle visual cortex. This commonality in function is remarkable given the differences in structure between mammalian neocortex and the ancestral cortex of reptiles. For instance, turtle visual cortex is comprised of three layers similar to mammalian piriform cortex and hippocampus [37–39]. Together with studies of mammalian brains, our results suggest that the detection-discrimination trade-off either evolved independently for turtles and mammals or has been evolutionarily preserved for hundreds of millions of years with origins as early as the emergence of amniotes.

A simple interpretation for why the detection-discrimination trade-off might occur arises from considering some basic ethological aspects of how we interact with the environment. When a new object or feature of the environment is first encountered, the first job of our visual system is to detect its presence. A likely second step is to examine and discriminate the finer details of the newly-detected thing. The utility of this temporal sequence—detection followed by discrimination–suggests an explanation for our findings in the turtle visual cortex as well as similar findings in mammalian somatosensory cortex [4–7]. Moreover, our results suggest that this detection-discrimination sequence is facilitated by the flexibility in cortical state that comes of operating near criticality.

Although our measurements were done in cortex, it is also likely that retinal and thalamic adaptation contribute to our results. Our model suggests that short-term depression among thalamocortical and corticocortical synapses may be sufficient to explain our results, but future experiments are required to determine the relative importance of adaptation in different parts of the visual system. Indeed, detection and discrimination may relate differently at different stages of the visual system.

Our modeling focused on synaptic depression as the relevant adaptive mechanism to parsimoniously explain our results. However, other adaptive mechanisms could also play roles. Indeed, synaptic facilitation could result in a strong transient response similar to that we observed [40]. Differing time courses of excitatory and inhibitory response to thalamocortical input could also be relevant [41,42]. Further study is required to pinpoint which possible mechanisms contribute to our observations.

Our study focused on the adaptation that follows the onset of a sensory stimulus. However, it is also well established (at least in mammals) that ongoing activity without sensory input can manifest as bouts of intense network activity (e.g. up-states [43] and neuronal avalanches [26]). Our results suggest that adaptive changes during such internal activity may also incur a trade-off between discrimination and detection. This trade-off may help to explain how response to sensory input is modulated by ongoing activity [43–45].

We designed our visual stimulus to cause a very clear and reliable time course of adaptation—an abrupt transition from no stimulus to a rather intense, dynamic movie. However, in reality, such an intense change is rarely part of naturalistic visual input. More realistically, the visual system is constantly receiving input; the “movie” does not turn off until we close our eyes. Thus, for realistic visual input it may be unusual for detection and discrimination to reach the extremes we observed during the transient onset response. Nonetheless, our results do have practical implications for the countless experimental studies in which a visual stimulus is presented following a black screen. For sustained natural visual input, our results suggest that the visual cortex spends most of its time near a scale-free dynamical regime, like the visually-driven steady state period we discuss above.

Our work focused on two specific aspects of sensory function—detection and discrimination. However, the computational repertoire of sensory cortex is certainly broader than just detection and discrimination. Different functions may require the underlying cortical network to be tuned differently. For instance, sensory dynamic range has been shown to be maximized at criticality [34,46,47]. Other functions such as oscillatory binding and information transmission across cortical regions [48,49] may benefit from synchronous (perhaps supercritical) dynamics, while object representation has been suggested to benefit from asynchronous (perhaps subcritical) dynamics [22]. Our results demonstrate a clear case of switching between competing computational properties depending on context. We expect future studies to build upon our findings to obtain a more comprehensive understanding of the computational versatility of the cortex.

## Materials and methods

### *Ex vivo* eye-attached whole-brain preparation

All procedures were approved by Washington University’s and University of Arkansas’ Institutional Animal Care and Use Committees and conform to the guidelines of the National Institutes of Health on the Care and Use of Laboratory Animals. Animal use protocol numbers were (20150248 for Wash U, and 16041 for U Ark) Adult red-eared turtles (n = 14, Trachemys scripta elegans, 150–200 g weight, 12–15 cm carapace length) were studied. Following anesthesia (Propofol 10 mg/kg) and decapitation, we surgically removed the brain, optic nerves, and eyes, from the cranium [16,50]. One eye was hemisected and drained, thus exposing the retina for visual stimulation; the other eye was removed. Two cuts allowed the cortex to be unfolded, exposing the ventricular surface, thus facilitating the subsequent insertion of the microelectrode array. The eye and the brain were continuously perfused with artificial cerebrospinal fluid (in mM; 85 NaCl, 2 KCl, 2 MgCl2, 45 Na HCO3, 20 D glucose, and 3 CaCl2 bubbled with 95% O2 and 5% CO2), adjusted to pH 7.4 at room temperature. Recordings began 2–3 hrs after induction of anesthesia.

### Microelectrode array measurements

Using a micromanipulator (Sutter, MP-285), we inserted a microelectrode array into the visual cortex. The array was comprised of a three dimensional grid of electrodes (4x4x8 grid, 16 shanks, 8 electrodes per shank, 300 μm inter shank spacing, 100 μm interelectrode spacing on each shank, Neuronexus). We analyzed data from every other electrode along each shank to avoid sampling redundant LFP. This included 48 electrodes in total. The array was inserted into visual cortex to a depth such that electrodes spanned the cortex from the ventricular to the dorsal surface. We recorded wideband (0.7 Hz– 15 kHz) extracellular voltages relative to a silver chloride pellet electrode in the bath at 30 kHz sample rate (Blackrock Microsystems, Cerebus). With post-processing filtering we extracted local field potential (LFP, band-pass 5–100 Hz) and multi-unit activity (MUA, band-pass 100–1000 Hz). MUA spike times were defined as the times of negative peaks that surpassed a -3 SD threshold.

### Visual stimuli

Visual stimuli were created by a computer and delivered with a miniature video projector (Aaxa Technologies, P4X Pico Projector). The projector image was focused onto the retina with additional lenses (Fig 2a). The mean light intensity (irradiance) at the retina was 1 W/m^2^.

For our stimulus detection measurements, the stimulation consisted of a transition from darkness to one of two types of grayscale movie (5 s in duration). One was the first 5 s of a ‘motion-enhanced’ movie [51], the other was a spatiotemporally phase-shuffled version of this movie. We also tested a transition from darkness to a static, uniform gray visual field.

For stimulus discrimination, the same movies were considered as “background” stimulation, upon which we added 1 of 4 different foreground stimuli. The foreground stimuli were a circular patch (dot) of uniform color ranging from gray to red in linear steps in RGB space. The dot spanned ¼ of the visual field centered in the middle of the visual field. The dot was presented for 30 ms either during the transient period (300 ms after background onset) or during the steady state period (4 s after background onset).

### Avalanche analysis

The first step of avalanche detection was to compute the standard deviation of every LFP trace. Next we defined an *‘LFP peak’* as a period of time during which an LFP trace fluctuates beyond 3 standard deviations, due to either a positive or negative deflection (Fig 2b). For each LFP peak, we determined the time of its extreme value and the identity of the channel on which it was recorded. An avalanche was defined as a spatiotemporal cluster of consecutive LFP peaks with inter-peak intervals not exceeding a temporal threshold ΔT (channel information does not play a role in avalanche definition). For each turtle, ΔT was chosen to be the average inter-peak interval (<IPI>, inverse of population LFP peak rate), which was 19.3 ± 7.3 ms. The size of an avalanche was defined as the number of LFP peaks comprising the avalanche. Avalanches were grouped and analyzed separately depending on whether they occurred during the transient period or visually-driven baseline or steady state periods.

### Power law fitting, fit quality *q*, and deviation *δ*

For avalanche size distributions in the baseline period, we used previously developed maximum likelihood fitting methods [16] to fit a truncated power law (truncated at both the head and tail). The fitting function was $f(S)=S^{-\tau }{\left(\sum_{x=x_{0}}^{x_{M}}x^{-\tau }\right)}^{-1}$, where the maximum size *x*_*M*_ was assumed to be the largest observed avalanches size. The minimum size *x*_0_ and the exponent *τ* were fitting parameters. Minimum values for *x*_0_ were tried increasing from 0, but only up to the point when the fitted power law matches the data well enough to have a Kolmogorov-Smirnov statistic $KS<1/\sqrt{N_{samp}}$, where *N*_*samp*_ is the number of avalanches comprising the dataset as established in previous work [16].

After finding the best-fit power law, the next step was to assess goodness-of-fit *q* [27,52]. We compared the experimental data to 1000 surrogate data sets drawn from the best-fit power law distribution with the same number of samples as the experimental data set. The deviation between the surrogate data sets and a perfect power law was quantified with the *KS* statistic. The quality *q* of the power law fit was defined as the fraction of these surrogate *KS* statistics which were greater than the *KS* statistic for the experimental data. A very conservative criterion for judging the data to be power law distributed is *q*>0.1. This is demonstrated visually in Fig 2e by plotting the experimental distribution over a green outlined region which delineates the 5–95 percentiles of the surrogate data sets.

We characterized changes in network dynamics with the measure δ. For the transient period δ was defined as the deviation between the baseline avalanche size distribution and the distribution of avalanches that occurred during the first second following stimulus onset. Similarly, for the “steady-state” period, δ is the deviation between the baseline distribution and the distribution of avalanches that occurred between 4–5 s following stimulus onset. To compute δ, we followed previously developed methods [16,47], first recasting the two compared distributions as cumulative distribution functions (CDF). Next, we took the mean of 10 differences between the two compared CDFs. The 10 differences were spaced logarithmically between minimum and maximum observed avalanche sizes. Thus, the range of possible δ is -1 to 1, with negative δ indicating a tendency for small-scale dynamics, positive δ indicating a tendency for large-scale dynamics, and δ = 0 indicating scale-free dynamics. In contrast, a Kolmogorov-Smirnov statistic is defined as the absolute value of the single largest difference between two cumulative distributions.

### I(R;S) and adjusting for low sample bias

The mutual information calculations used to quantify detection and discrimination were adjusted to correct for potential finite sampling bias. We followed well established, non-parametric methods [53,54], sometimes referred to as ‘bootstrap correction’ [55]. More specifically, the naïve uncorrected mutual information was reduced by subtracting a surrogate ‘noise’ mutual information I(R;S_shuff_) obtained by recomputing mutual information with the order of stimuli randomized. I(R;S_shuff_) was computed for 100 independent randomizations of the stimuli order; error bars in Figs 3 and 4 reflect variability across these 100 randomizations. When the true mutual information is high, this correction scheme provides a conservative estimate, with a slight bias towards underestimating the true mutual information (S1 Text). However, the correction is less biased and particularly important when the true mutual information is low (S1 Text). Our primary conclusions–different discrimination and detection during the transient response compared with the steady-state response–would likely be even stronger if we were able to do more repetitions of the stimuli. To further mitigate low sampling bias, we also reduced the dimensionality of LFP and MUA response by categorizing each response into one of four bins defined by the following bin edges: 0, R_20_ + ΔR/4, R_20_ + ΔR/2, R_20_ + 3ΔR/4, R_max_. Here R_20_ is defined as the 20^th^ quantile of all responses for a given time period (e.g. for all transient responses or all steady state responses), ΔR is the differences between the 80^th^ and 20^th^ quantiles, and R_max_ is the largest response.

### Computational model

*N* = 1000 all-to-all connected binary neurons received input from outside the network. The ‘strength’ of the synapse from neuron *j* onto neuron *i* at time *t* is determined by the corresponding element of the synaptic weight matrix *W*_*ij*_(*t*). 20% of neurons are inhibitory, i.e. with negative entries in the weight matrix. Ω_*i*_(*t*) is the strength of the input synapse onto neuron *i* (all excitatory). The binary state *s*_*i*_(*t*+1) of neuron *i* (*s* = 0 inactive, *s* = 1 spiking) is determined probabilistically based on the sum *p*(*t*+1) of its inputs $p(t+1)=\Omega_{i}(t)\sigma_{i}(t)+\sum_{j=1}^{N}W_{ij}(t)s_{j}(t)$. If 0 < *p* < 1, then the neuron fires with probability *p*. If *p* ≥1, then the neuron fires with probability 1. If *p* ≤ 0, then the neuron does not fire. Time is discrete and state updates are synchronous. The input *σ*_*i*_(*t*) from the *i*th input synapse is binary (1 with probability *r(t)*). The dynanmics of *r(t)*, define the stimulus in the model as discussed further below. The update rules for synaptic dynamics are

$$W_{ij}(t+1)=W_{ij}(t)+\tau_{r}{}^{-1}\left(W_{ij}^{o}-W_{ij}(t)\right)-\tau_{d}{}^{-1}W_{ij}(t)s_{j}(t)$$

$$\Omega_{i}(t+1)=\Omega_{i}(t)+\tau_{r}{}^{-1}\left(\Omega_{i}^{o}-\Omega_{i}(t)\right)-\tau_{d}{}^{-1}\Omega_{i}(t)\sigma_{i}(t).$$

The default weight matrix $W_{ij}^{o}$ was constructed such that its largest eigenvalue Λ_0_ has absolute value equal to 1.03 (this results in synaptic strengths near 1.03/N on average) [16,56]. Default input synaptic strengths $\Omega_{i}^{o}$ were 0.02. Synapses depress with a time constant of *τ*_*d*_ = 40 time steps following a presynaptic spike and recover exponentially with a time constant of *τ*_*r*_ = 400 time steps. These parameter choices were made based on previous work with this model [16] and with the goal of reproducing our experimental results.

To model the background stimulus used for studying detection, the onset of stimulation is modelled as a step increase from a constant low level (*r* = 5x10^-4^) to a higher level that slowly fluctuates as *r*(*t*) = 0.1 +0.075 sin(0.0075*t* + 2*πφ*), where φ was varied across trials (in linear steps from 0 on trial 1, to 1 on trial 80). This background stimulus is delivered to half of the neurons. To model the brief foreground stimulus used to study discrimination, we set r(t) = 0.05, 0.1, 0.2 or 0.4 (to mimic the 4 levels of foreground stimulus (red dots) in the experiments) for 30 model time steps for all neurons before returning to the background stimulus.

In the model, an avalanche is initiated by external input. Upon reaching a time step with no active neurons, the avalanche is considered to be ended. We simulated 80 trials of step increase in input for detection and 80 trials for discrimination (20 for each foreground stimulus). Each trial consisted of 2500 time steps (1 time step can be interpreted to be approximately 1 ms). The background stimulus onset was at time 500. The foreground stimulus was presented between times 530 and 560 (transient) or between times 2000 and 2030 (steady state).

## Supporting information

S1 Text Interpreting mutual information considering low sample bias. We demonstrate how our experimental results depend on the number of repeated trials for the visual stimulation and how such finite sampling is expected in theory to affect estimates of mutual information.
(DOCX)
