# Supplementary material for: Adaptation towards scale-free dynamics improves cortical stimulus discrimination at the cost of reduced detection
Source: PLoS Comput Biol. 2017 May 30;13(5):e1005574. doi: 10.1371/journal.pcbi.1005574 (PMC5469508; doi:10.1371/journal.pcbi.1005574)
Supplement: S1 Text — We demonstrate how our experimental results depend on the number of repeated trials for the visual stimulation and how such finite sampling is expected in theory to affect estimates of mutual information. (DOCX) [file pcbi.1005574.s001.docx]

**S1 Text**

**Interpreting mutual information considering low sample bias.**

Our primary conclusions depend on estimating mutual information between stimuli and responses. For instance, we conclude that discrimination of the 4 red dot stimuli is higher during the steady-state response than in the transient response. However, as in any experiment, we cannot repeat the 4 stimuli an infinite number of times. How do such finite sampling limitations affect estimates of mutual information? This topic has been studied extensively (for example [1]). In general, if no corrections are made, the mutual information will be overestimated. Overestimation errors become extreme for very few stimulus repetitions and when the actual mutual information is low. To mitigate these errors, the simplest scheme is to simply subtract a so-called ‘bootstrap’ correction. The correction is obtained by recomputing the mutual information with the stimuli in randomized order relative to the responses. This is repeated many times, for 100 independent randomizations. The average of the differences between the measured mutual information and these 100 bootstrap corrections is the quantity we report in the main manuscript (called either ‘discrimination’ or ‘detection’). Unlike mutual information without any bias correction, these corrected values can be positive or negative. Negative values typically occur when the mutual information is low. In Figure S1, we show explicitly for 3 example turtles how these corrections depend on the number of stimulus repetitions, by systematically considering subsets with differing numbers of stimulus presentations from the actual experimentally presented stimuli.

Moreover, we show a similar analysis of three artificial “theoretical” datasets with far more “repetitions” which shows how these corrections work and how they compare to the real mutual information. The theoretical datasets were generated as follows. The “stimulus” set was simply defined as the sequence 1, 2, 3, 4, repeated many times. Two different response sets were defined in two steps. First, the stimulus set was modified by adding Gaussian noise with either 0.5 or 1.5 standard deviation, for the “high information” or “medium information” cases respectively. Then the response set was binned according to the same strategy described in the Materials and Methods section for rate-based response. For the “zero information” case, the response set was Gaussian noise completely unrelated to the stimulus, binned into 4 categories.

Based on comparing our theoretical datasets to our experimental examples, we expect that our experimental estimates are accurate within about 10-20%, tending to underestimate the true information. More importantly, the primary conclusions of our paper are robust when considering the biases caused by limited stimulus repetitions. More stimulus presentations would likely raise our estimates of mutual information more for cases with high mutual information compared to cases with low mutual information. Thus, more stimulus presentations are likely to make differences in mutual information across cases more extreme, further strengthening our conclusions.


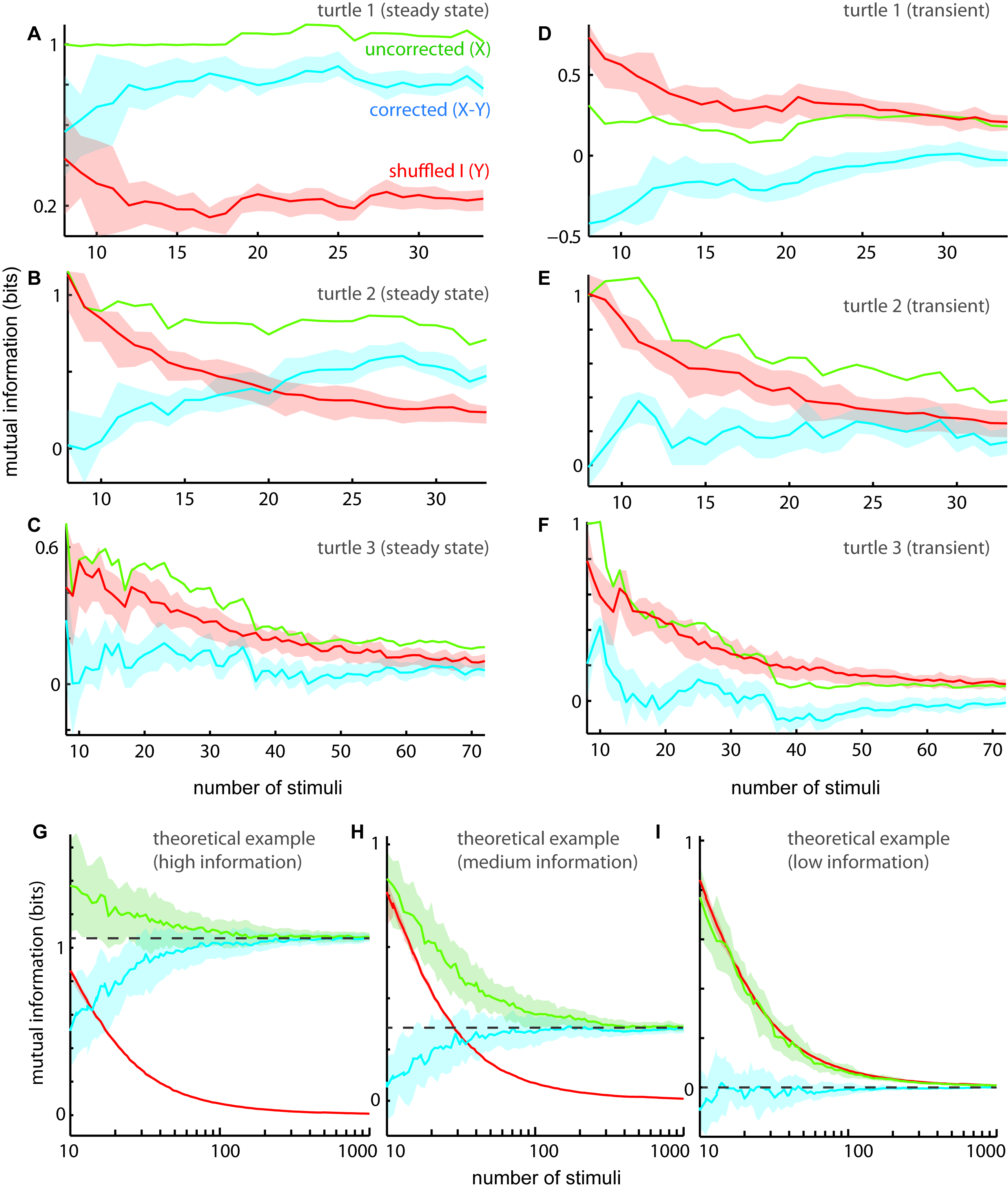
 **Fig S1. How mutual information depends on the number of stimulus trials.** In panels A-F, the green curve represents a raw, uncorrected mutual information between stimulus (4 red dot levels) and response (rate-based, LFP) from our experiments. The red line represents a control mutual information obtained by randomizing the order of stimuli relative to the order of responses. The pink shaded area delineates quartiles for 100 such controls. The blue line is our estimate of mutual information obtained by subtracting the red line from the green. The right-most value of the blue line is the value of ‘discrimination’ presented in Fig 3 of the main manuscript. Panels A-C are for steady state response measurements in three example turtles. Panels D-F are for transient response measurements in the same three turtles. Panels G-I represent artifical theoretical datasets with up to 1000 repetitions of stimuli. For high numbers of stimuli, corrections are unnecessary and the true value of mutual information is clear (dashed line). The shaded quartiles represent variation over an ensemble of 50 different theoretical datasets. Note that the corrected mutual information underestimates the true value. This underestimation is less extreme when the true mutual information is lower.
